# Parkin depletion prevents the age-related alterations in the FGF21 system and the decline in white adipose tissue thermogenic function in mice

**DOI:** 10.1007/s13105-023-00977-x

**Published:** 2023-11-02

**Authors:** Alejandro Delgado-Anglés, Albert Blasco-Roset, Francisco J. Godoy-Nieto, Montserrat Cairó, Francesc Villarroya, Marta Giralt, Joan Villarroya

**Affiliations:** 1 Departament de Bioquímica i Biomedicina Molecular and Institut de Biomedicina, Universitat de Barcelona, Barcelona, Spain; 2 Centro de Investigación Biomédica en Red “Fisiopatología de la Obesidad y Nutrición”, Madrid, Spain; 3 Institut de Recerca Hospital Sant Joan de Déu, Barcelona, Spain

**Keywords:** Adipose tissue, Parkin, Aging, Fibroblast growth factor-21, Metabolism

## Abstract

Parkin is an ubiquitin‐E3 ligase that is involved in cellular mitophagy and was recently shown to contribute to controlling adipose tissue thermogenic plasticity. We found that Parkin expression is induced in brown (BAT) and white (WAT) adipose tissues of aged mice. We determined the potential role of Parkin in the aging-associated decline in the thermogenic capacity of adipose tissues by analyzing subcutaneous WAT, interscapular BAT, and systemic metabolic and physiological parameters in young (5 month-old) and aged (16 month-old) mice with targeted invalidation of the Parkin (*Park2*) gene, and their wild-type littermates. Our data indicate that suppression of Parkin prevented adipose accretion, increased energy expenditure and improved the systemic metabolic derangements, such as insulin resistance, seen in aged mice. This was associated with maintenance of browning and reduction of the age-associated induction of inflammation in subcutaneous WAT. BAT in aged mice was much less affected by Parkin gene invalidation. Such protection was associated with a dramatic prevention of the age-associated induction of fibroblast growth factor-21 (FGF21) levels in aged Parkin-invalidated mice. This was associated with a parallel reduction in FGF21 gene expression in adipose tissues and liver in aged Parkin-invalidated mice. Additionally, Parkin invalidation prevented the protein down-regulation of β-Klotho (a key co-receptor mediating FGF21 responsiveness in tissues) in aged adipose tissues. We conclude that Parkin down-regulation leads to improved systemic metabolism in aged mice, in association with maintenance of adipose tissue browning and FGF21 system functionality.

**Supplementary Information:**

The online version contains supplementary material available at 10.1007/s13105-023-00977-x.

## Introduction

The thermogenic capacity of brown adipose tissue (BAT) and beige adipose tissue resulting from the “browning” of white fat (WAT) declines with aging in humans and experimental rodent models [37]. This decline is considered to contribute to the propensity of elderly individuals to develop metabolic diseases, from overweight to type 2 diabetes and dyslipidemia. The mechanisms leading to the age-associated functional decline in thermogenic adipose tissue are poorly understood despite the obvious biomedical interest in preventing this shift.

Fibroblast growth factor 21 (FGF21) is a member of the endocrine FGF subfamily that is produced primarily in the liver and adipose tissues. Treatment with FGF21 ameliorates metabolic disorders such as insulin resistance, dyslipidemia and obesity in mice [6, 19], and treatment of human volunteers with FGF21 analogs has shown beneficial effects on metabolic disorders ranging from obesity and type 2 diabetes to non-alcoholic liver diseases [31]. The capacity of FGF21 to activate BAT thermogenesis and promote the “beiging” of WAT [7, 11, 17] is considered to contribute to its beneficial metabolic effects. The actions of FGF21 are mediated through interactions with FGF receptors on the cell surface and require the coreceptor, β-klotho [27]. In fact, the expression of β-klotho determines the specific responsiveness of cells to FGF21. Transgenic overexpression of FGF21 in mice, leading to high levels of circulating FGF21, was reported to extend the lifespan [36], prompting researchers to propose that FGF21 is a pro-longevity endocrine factor. Consistent with this, FGF21 was reported to critically mediate the beneficial effects on lifespan elicited by dietary protein restriction [16]. On the other hand, FGF21 levels in blood increase with age in rodents and humans [12, 15]. This could seem somewhat paradoxical considering the healthy metabolic effects expected from high FGF21 levels and the tendency of metabolic alterations to appear with age. It has been hypothesized that the age-related increases in FGF21 are related to the appearance of an FGF21-resistant state, as has been proposed to occur in metabolic diseases such as obesity and type 2 diabetes [10, 29]. However, recent data from aged humans are not compatible with this proposal [33], and low levels of FGF21 have been related to healthy aging in centenarians [30].

Recent work revealed that Parkin contributes to controlling BAT activity and the browning of WAT. Parkin is an ubiquitin‐E3 ligase that acts as a key component of the cellular machinery for mitophagy and apoptosis. The name comes from the identification of mutations in the Parkin-encoding gene as being responsible for early-onset forms of Parkinson disease, a neurodegenerative condition that is usually associated with aging [8]. The actions of Parkin are reported to be reciprocally related to the thermogenic activity of adipose tissues. Parkin is required for the adaptive down-regulation of BAT thermogenic activity [1, 27] and the induction of “whitening” in beige adipose tissue [20, 32] in response to cessation of environmental thermal challenges, likely through its role in promoting the degradation of components of the mitochondrial thermogenic machinery.

Here we report that Parkin levels in adipose tissue increase with aging which led us to hypothesize that Parkin could be involved in the aging-associated down-regulation of the thermogenic properties of adipose tissues. Therefore, we studied the impact of Parkin invalidation on thermogenic adipose tissues in aged mice and characterized the key alterations elicited by Parkin invalidation on the age-induced decline in WAT browning and FGF21 homeostasis.

## Materials and methods

### Mouse studies

For studies on changes in Parkin expression across aging, male C57/BL6 mice, obtained from Envigo, were used. For studies on the effects of Parkin gene invalidation, Parkin‐KO (B6.129S4‐Park2tm1Shn/J) mice were obtained from Jackson Laboratories and wild‐type (WT) littermates were used as controls. Mice were housed at 22 °C under a 12-h light cycle with free access to water. Mice were fed a standard diet (3.1 kcal/g, 24% protein, 58% carbohydrates, 18% fat; Teklad, Envigo). Male mice were studied at ages of 5 months (adult) and 16 months (aged). The volume of consumed oxygen and CO2 released was determined using an Oxylet system and analyzed with the Oxylet Metabolism 3.0 software (Harvard Apparatus). Oxygen consumption measurements, calculations of metabolic efficiency, glucose tolerance tests (GTT), and insulin tolerance tests (ITT) were performed in the 3-week period before sacrifice. For GTT, glucose in aqueous solution was administered intraperitoneally (2.5 g glucose/kg) to overnight-fasted mice, blood was obtained from the tail 0, 15, 30, 60, 90, 120, and 150 min after glucose injection, and glycemia was measured. For ITT, insulin (Actrapid; Novo Nordisk Pharma) in saline solution was administered intraperitoneally (0.75 UI/kg) to 4-h-starved mice, and glycemia in blood obtained from the tail was measured 0, 15, 30, 45, and 60 min after glucose injection. Areas under the curve were calculated from individual curves of glycemia changes after GTT and ITT.

After sacrifice, interscapular BAT (iBAT), inguinal WAT (iWAT), epididymal adipose tissue (eWAT), liver, heart and tibia were dissected, and blood was collected in heparinized tubes to obtain plasma. All experiments were performed in accordance with European Community Council Directive 86/609/EEC, and the experiments and numbers of animals to be used were approved by the Institutional Animal Care and Use Committee of the University of Barcelona.

### Analytical procedures for blood and plasma samples

Glucose and triglyceride levels in blood were measured using Accutrend Technology (Roche Diagnostics). Plasma FGF21 was quantified using an enzyme-linked immunosorbent assay (ELISA) (RD291108200R, BioVendor). Plasma insulin and leptin levels were quantified using a Multiplex system (MADKMAG‐HK, Merck Millipore).

### Optical microscopy

For hematoxylin and eosin (H&E) staining, tissue samples were fixed overnight in 4% paraformaldehyde, paraffin‐embedded and processed in a microtome. Tissue sections of 4 μm thickness were mounted onto glass slides, dewaxed, rehydrated, stained with H&E, dehydrated again in a graded ethanol/xylene series and sealed with DPX and a coverslip, according to standard procedures. Adipocyte size was analyzed in sections of inguinal adipose tissue; digital images were captured using an Olympus DP70 digital camera (Olympus) attached to a bright-field light microscope (Nikon Eclipse 90i Upright Microscope). At least four fields of view were analyzed for each sample (*n* = 3 samples per genotype per timepoint). All images were acquired under the same conditions at 200 × magnification. Adipocyte sizes were measured using the open-source imaging program, Fiji (a distribution of ImageJ), with the semi-automated plug-in, Adiposoft.

### RNA isolation and quantitative real‐time PCR

RNA was extracted from adipose tissues and liver using a NucleoSpin RNA column kit (Macherey‐Nagel). Reverse transcription of 0.5 μg of total RNA in a total reaction volume of 20 µL was performed using a high-capacity complementary DNA (cDNA) kit. Samples were systematically checked for null amplification in the absence of reverse transcriptase. qRT-PCR was performed using the appropriate TaqMan probes (Supplemental Table 1); each 25 µL reaction mixture contained 1 µL cDNA, 12.5 µL TaqMan Universal PCR Master Mix (Thermo Fisher Scientific) and 250 nM probes. The cDNA level of each gene of interest was normalized to that of a housekeeping reference gene (*Ppia* mRNA). The comparative CT (2^−ΔCt^) method was applied for normalization, according to the manufacturer’s instructions. Main results were checked using the 18S rRNA as a second independent housekeeping gene.

### Western blotting

Tissue extracts were prepared by homogenization in a buffer consisting of 20 mM Tris–HCl (pH 7.4), 1 mM EDTA, 1 mM EGTA, 1% Triton X‐100, a protease inhibitor cocktail (Roche Diagnostics), 2 mM sodium orthovanadate, and 10 mM β‐glycerophosphate. The total protein content was measured using a Pierce™ BCA Protein Assay Kit (Fisher Scientific). Equal amounts of protein (30 µg) were separated by sodium dodecylsulfate-polyacrylamide gel electrophoresis (SDS-PAGE) on 12% or 15% gradient gels and electrotransferred onto Immobilon‐P PVDF (polyvinylidene difluoride) membranes (GE Healthcare). The membranes were incubated with primary antibodies specific for Parkin (2132, Cell Signaling Technology), β-klotho (ab76356, Abcam) and tyrosine hydroxylase (ab152, Merck Millipore), and then with horseradish peroxidase (HRP)‐conjugated anti‐mouse IgG (170‐6516, Bio‐Rad) or anti‐rabbit IgG (sc‐2004, Santa Cruz), as appropriate. Signals were detected using a chemiluminescence‐HRP substrate (WBKLS0100, Merck). Membranes were stained with Ponceau to normalize the amount of protein loaded. Densitometric analyses of digitalized images were performed using the Fiji software (ImageJ).

### Statistical analysis

Statistical significance was assessed using the two‐tailed unpaired Student's t‐test, one-way ANOVA or two‐way ANOVA followed by Tukey's post-hoc test, all of which were applied using GraphPad statistical software (GraphPad Software). Discrepancies among standard deviations of experimental groups were analyzed with the F‐test. Welch's correction was applied whenever unequal variances were detected. Exact numbers of replicates are shown in each Figure legend. Outliers were detected using Grubbs’ test and removed prior to analysis of significance. Statistical significance was set at *P* < 0.05, and the underlying assumptions for validity were assessed for all tests. Data are expressed as means ± standard error of the mean (SEM).

## Results

1. Parkin expression is up-regulated in aged brown and white adipose tissues

**Fig. 1:**
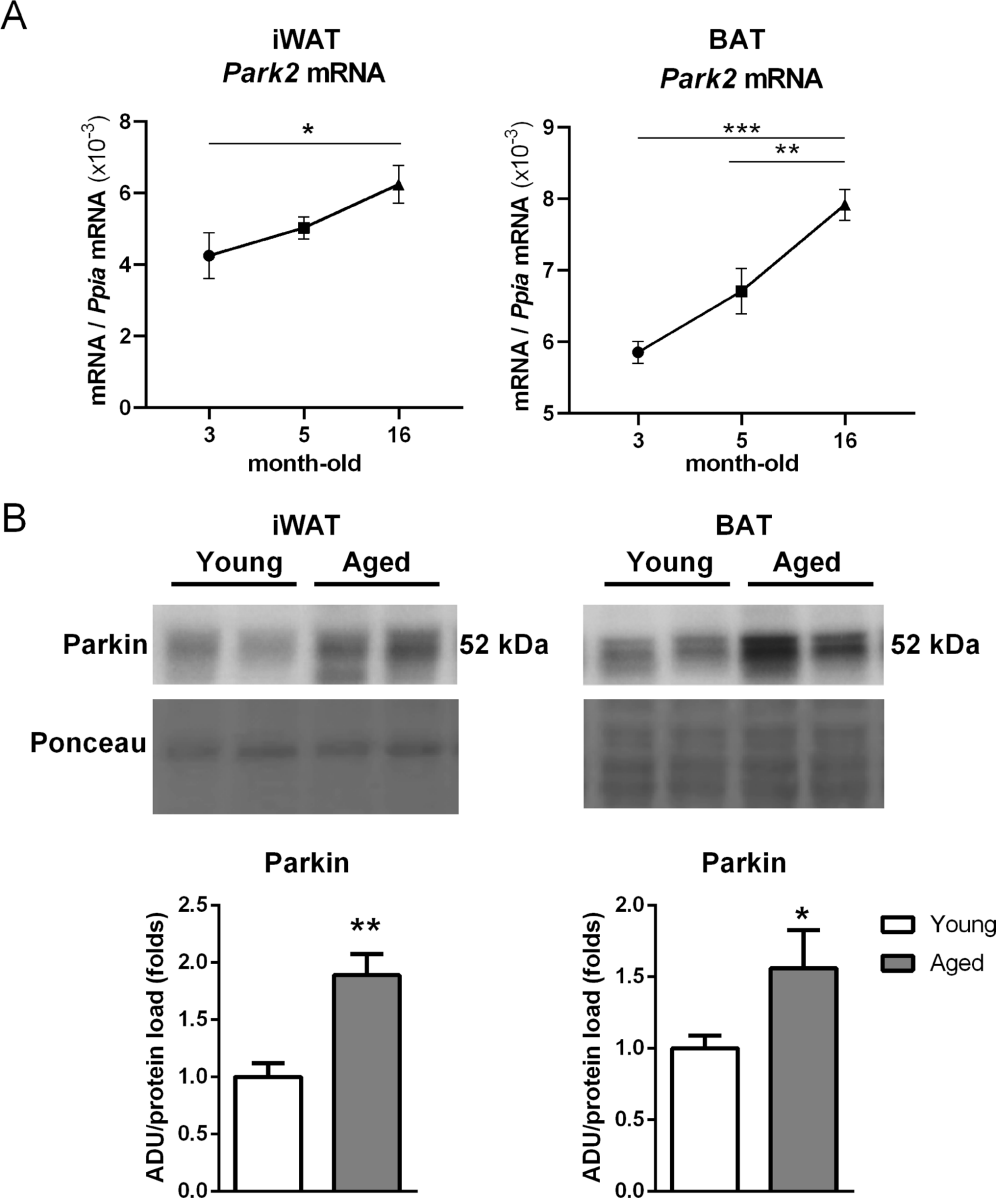
Gene expression and protein levels of Parkin in subcutenous white and brown adipose tissues upon aging. (**A**) Gene expression levels of *Park2* (Parkin gene) in inguinal white adipose tissue (iWAT) and brown adipose tissue (BAT) in mice at 3, 5 and 16 months of age. (**B**) Representative immunoblot (top) and quantification of Parkin protein levels (bottom) in iWAT and BAT from young (5 month-old) versus aged (16 month-old) mice. *N* = 3–9. The bars represent means ± SEM (*p < 0.05, **p < 0.01, ***p < 0.001 when comparing young versus aged mice)

Parkin gene expression was determined in iWAT and BAT across aging in mice. Parkin mRNA levels increased progressively from 3 to 16 months of age (Fig. 1A). Parkin protein levels were up-regulated in iWAT and BAT from aged mice (16-month-old) relative to young (5-month-old) mice (Fig. 1B).

2. Parkin gene invalidation reduces age-dependent adiposity and associated metabolic derangements

To determine the role of Parkin in the aging of adipose tissues, we analyzed Parkin-KO mice in comparison with wild-type littermates in young adult (5-month-old) and aged (16-month-old) mice (Table 1). At 5 months of age, Parkin-KO mice showed a mild and non-significant trend toward decreased body weight and adipose depot mass compared to wild-type littermates. At 16 months of age, wild-type mice had significantly increased body weight, mostly due to fat accretion (massive enlargement of iWAT and eWAT) relative to young wild-type mice. These age-dependent changes were much weaker in 16-month-old Parkin-KO mice, which showed significantly lower body weight, massive reductions in the amounts of iWAT, eWAT, and iBAT, and even a reduction in liver weight, compared to the corresponding wild-type mice. These changes did not reflect any notable alteration in developmental growth, as evidenced by the lack of difference in tibia length between wild-type and Parkin-KO mice at 16 months of age.

**Table 1:** Metabolic effects of Parkin gene invalidation in aging mice

	Young		Aged	
	WT	KO	WT	KO
Body weight (g)	30,3 ± 0,7	27,4 ± 0,6	42,8 ± 1,0^###^	34,7 ± 0,8***
iWAT (mg)	209 ± 29	193 ± 32***	1162 ± 108^###^	**686 ± 86**^###^***
eWAT (mg)	415 ± 70	390 ± 55	1595 ± 72^###^	**1286 ± 106**^###^*
iBAT (mg)	84,7 ± 4,9	59,2 ± 2,0	154,0 ± 14,0^###^	**100,0 ± 9,4**^#^***
Liver (g)	1,32 ± 0,03	1,11 ± 0,05***	1,82 ± 0,09	1,23 ± 0,04^###^
Tibia length (mm)	17,8 ± 0,2	17,8 ± 0,3	18,4 ± 0,1	18,5 ± 0,1
Glucose (mg/dL)	138 ± 5	149 ± 4	182 ± 11^###^	158 ± 6
Insulin (pg/dL)	741 ± 154	797 ± 142	1657 ± 309^##^	588 ± 113**
Triglycerides (mg/dL)	183 ± 20	180 ± 11	161 ± 12	147 ± 14
Leptin (µg/dL)	1,4 ± 0,6	2,7 ± 0,7	8,7 ± 2,7^##^	4,8 ± 1,1
Food intake(kcal/day)	10,7 ± 0,2	9,78 ± 0,2	10,2 ± 0,3	8,35 ± 0,3***
Metabolic efficiency (g/MJ × 10^–1^)	18,2 ± 3,0	31,9 ± 6,1	2,34 ± 2,7^#^	1,9 ± 2,9^###^
VO_2_ (ml x min^−1^)	2,25 ± 0,06	2,28 ± 0,05	2,30 ± 0,04	**2,59** ± 0,07*
VO_2_ (ml x min^−1^)/(BW^0,75^)	0,18 ± 0,01	0,20 ± 0,01	0,14 ± 0,004^###^	0,20 ± 0,01***
RQ	0,86 ± 0,02	0,87 ± 0,02	0,87 ± 0,02	0,88 ± 0,02
GTT (AUC)	21,2 ± 0,7	26,4 ± 0,7	57,9 ± 7,2^#^	67,3 ± 4,4^#^
ITT (AUC)	5,78 ± 0,1	5,87 ± 0,7	9,70 ± 0,4^#^	7,34 ± 0,3*

Morphometric, energy balance-related parameters, tissue weight and circulating levels of metabolites and hormones in young and aged WT and Parkin-KO miceData are means ± s.e.m. (*N* = 4–18). *p < 0.05, **p < 0.01, ***p < 0.001 comparing wild-type (WT) versus Parkin-KO; #*p* < 0.05, ##*p* < 0.01, ###*p* < 0.001 aged versus young; Two-way ANOVA with Tukey’s post hoc test was used for comparisons. Statistical significance due to Parkin invalidation was highlighted in bold. Food intake and metabolic efficiency data correspond to the 3 week period before sacrifice. *iWAT* Inguinal white adipose tissue; *eWAT* Epididymal white adipose tissue. *iBAT* Interscapular brown adipose tissue. *BW* Body weight; *RQ* Respiratory quotient; *GTT* Glucose tolerance test. *ITT* Insulin tolerance test. *AUC* Area under the curve

Aged Parkin-KO mice showed systemic metabolic changes relative to aged wild-type mice (Table 1). Wild-type mice at 16 months of age showed higher glycemia, much higher insulin levels than 5-month-old wild-type mice, impaired glucose tolerance and reduced insulin sensitivity in the insulin tolerance test, indicating the appearance of insulin resistance with aging. Most of these alterations were prevented in aged Parkin-KO mice, which did not show basal hyperglycemia or hyperinsulinemia, showed improved insulin sensitivity test relative to aged wild-type mice, although the glucose tolerance was not improved. Triglyceride levels were unaltered in relation to age or genotype, whereas leptin levels were markedly increased at 16 months of age relative to 5 months of age; this alteration was more pronounced in wild-type mice than in Parkin-KO mice, likely reflecting the observed changes in the sizes of WAT depots.

We also observed significantly reduced food intake in 16 month-old Parkin-KO mice relative to wild-type mice. Calculation of metabolic efficiency indicated that the body weight accretion per ingested calorie was significantly lower in 16 month-old Parkin-KO mice than in wild-type mice, indicating that the reduced adiposity in aged Parkin-KO mice is likely to involve enhanced energy expenditure as an additional process contributing to the reduction in body weight. Accordingly, we found significantly higher levels of oxygen consumption, indicative of enhanced energy expenditure, in 16-month-old Parkin-KO mice relative to the corresponding wild-type mice (Table 1), whereas respiratory quotient was not altered by Parkin invalidation at any age. When oxygen consumption data were corrected per body weight, the impaired oxygen consumption associated with aging in wild-type mice was largely prevented in aged Parkin-KO mice.

3. Parkin invalidation partially prevents the age-dependent decline in WAT browning but does not alter BAT thermogenesis decline.

**Fig. 2:**
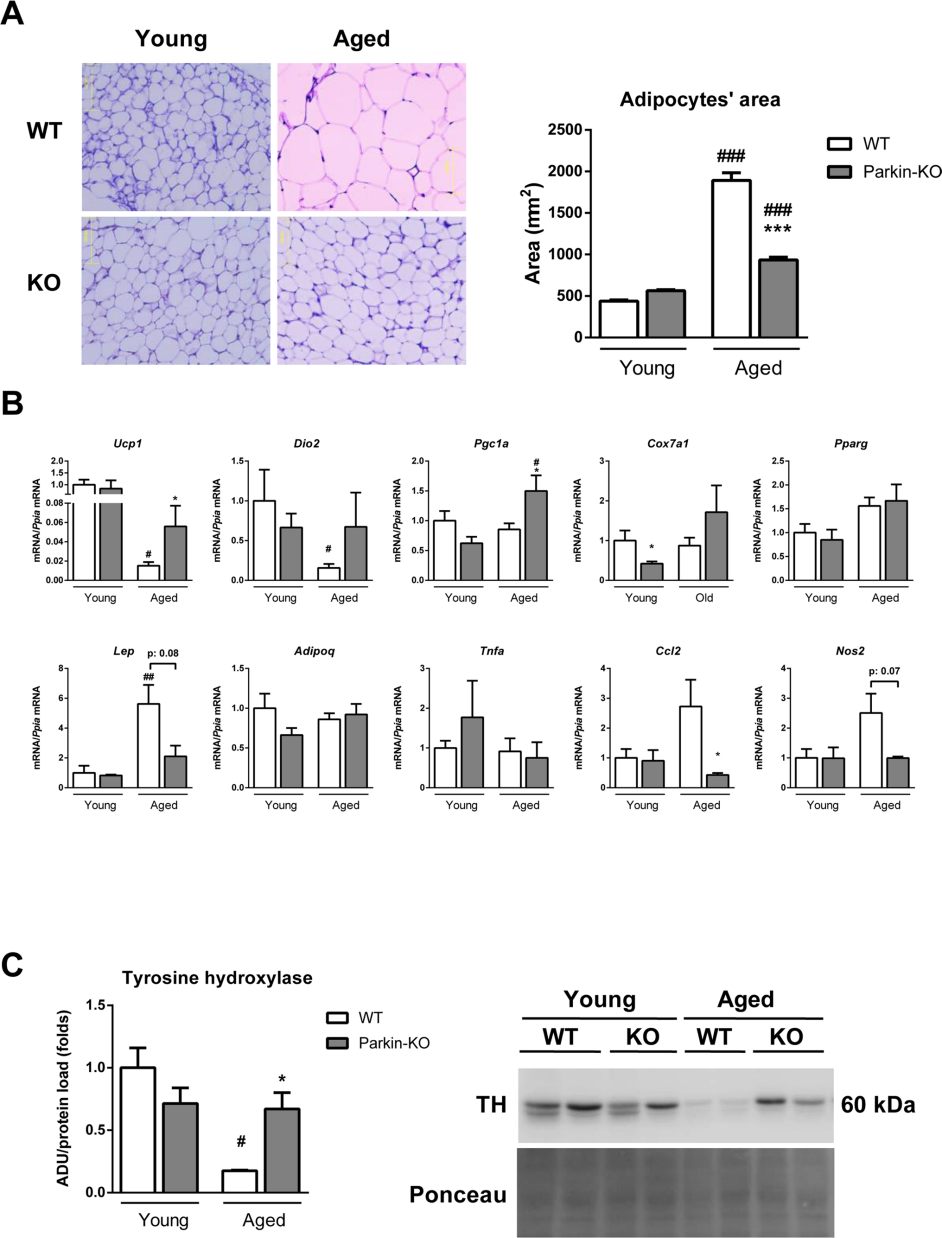
Adiposity and metabolic functionality markers in white adipose tissue from wild-type and Parkin knock-out young and aged mice. (**A**) Histological representative images (left) and quantification of white adipocytes area (right) from young (5 month-old) versus aged (16 month-old) wild-type and Parkin-KO mice. (**B**) Quantification of mRNA levels of markers related to browning (*Ucp1, Dio2, Pgc1a, Cox7a1*), adipose tissue function (*Pparg, Lep, Adipoq*), and inflammation (*Tnfa, Ccl2, Nos2*) in white adipose tissue from young versus aged wild-type and Parkin-KO mice. (**C**) Quantification and representative image of immunoblots for tyrosine hydroxylase protein in white adipose tissue samples from young versus aged wild-type and Parkin-KO mice. *N* = 3–7. The bars represent means ± SEM (*p < 0.05, ***p < 0.001 when comparing wild-type versus Parkin-KO mice; #*p* < 0.05, ##*p* < 0.01, ###*p* < 0.001 when comparing young versus aged mice)

Analysis of the microscopic morphology of iWAT revealed only minor differences between Parkin-KO and wild-type mice at 5 months of age. At 16 months of age, in contrast, white adipocytes were much smaller in iWAT from Parkin-KO mice compared to that of wild-type mice (Fig. 2A), revealing that Parkin invalidation protects mice from age-associated adipocyte hypertrophy. Gene expression analysis showed that wild-type mice experienced declines in the expression levels of marker genes for adipose browning, e.g. *Ucp1* (uncoupling protein 1), *Dio2* (type II iodothyronine 5’-deiodinase)*, Cox7a1* (cytochrome c oxidase subunit 7A1)*,* with aging, but this was totally or partially prevented in Parkin-KO mice. Expression of *Ucp1* and *Pgc1a* (PPARγ-coactivator-1α) were higher in iWAT from aged Parkin-KO mice relative to aged wild-type mice (Fig. 2B). The marked up-regulation in leptin gene expression due to aging was also prevented in Parkin-KO mice. The expression levels of genes related to inflammatory pathways, such as *Ccl2* (encoding chemokine (C–C motif) ligand 2) and *Nos2* (encoding nitric oxide synthase 2), were up-regulated in WAT from aged wild-type mice but not aged Parkin-KO mice (Fig. 2B). In contrast, the expression levels of genes involved in lipid metabolism, oxidative stress, ER stress and autophagy did not reveal relevant changes due to Parkin invalidation in aged mice (Supplemental Table 2). Moreover, the protein levels of tyrosine hydroxylase (TH), an indicator of the extent of sympathetic innervation at adipose tissue, were also reduced with aging in wild-type mice but not in Parkin-KO mice (Fig. 2C). Overall, these data indicate that the extent of WAT browning declines with aging, but Parkin gene invalidation prevents this decline. Moreover, WAT from aged Parkin-KO mice show a healthier phenotype associated with a leaner condition, as evidenced by reduced inflammation.

Analyses of microscopic morphology and gene expression patterns failed to reveal marked changes caused by Parkin invalidation in BAT from 16-month-old mice (Supplemental Fig. 1A). The expression of marker genes for thermogenic activity (e.g. *Ucp1, Pgc1a*) declined with aging similarly in wild-type and Parkin-KO mice (Supplemental Fig. 1B). The protein levels of TH were also similarly reduced in BAT from 16-month-old wild-type and Parkin-KO mice relative to 5-month-old wild-type and Parkin-KO mice (Supplemental Fig. 1C). Similarly unaltered gene expression pattern was found in the mRNA levels of genes encoding components of lipid metabolism, oxidative stress, ER stress, and autophagy in Parkin-KO mice at 5 or 16 months of age, relative to wild-type (Supplemental Table 3).

4. Parkin gene invalidation suppresses the aging-dependent inductions of FGF21 mRNA and protein levels while preventing the age-induced decline of β-klotho in adipose tissues.

**Fig. 3:**
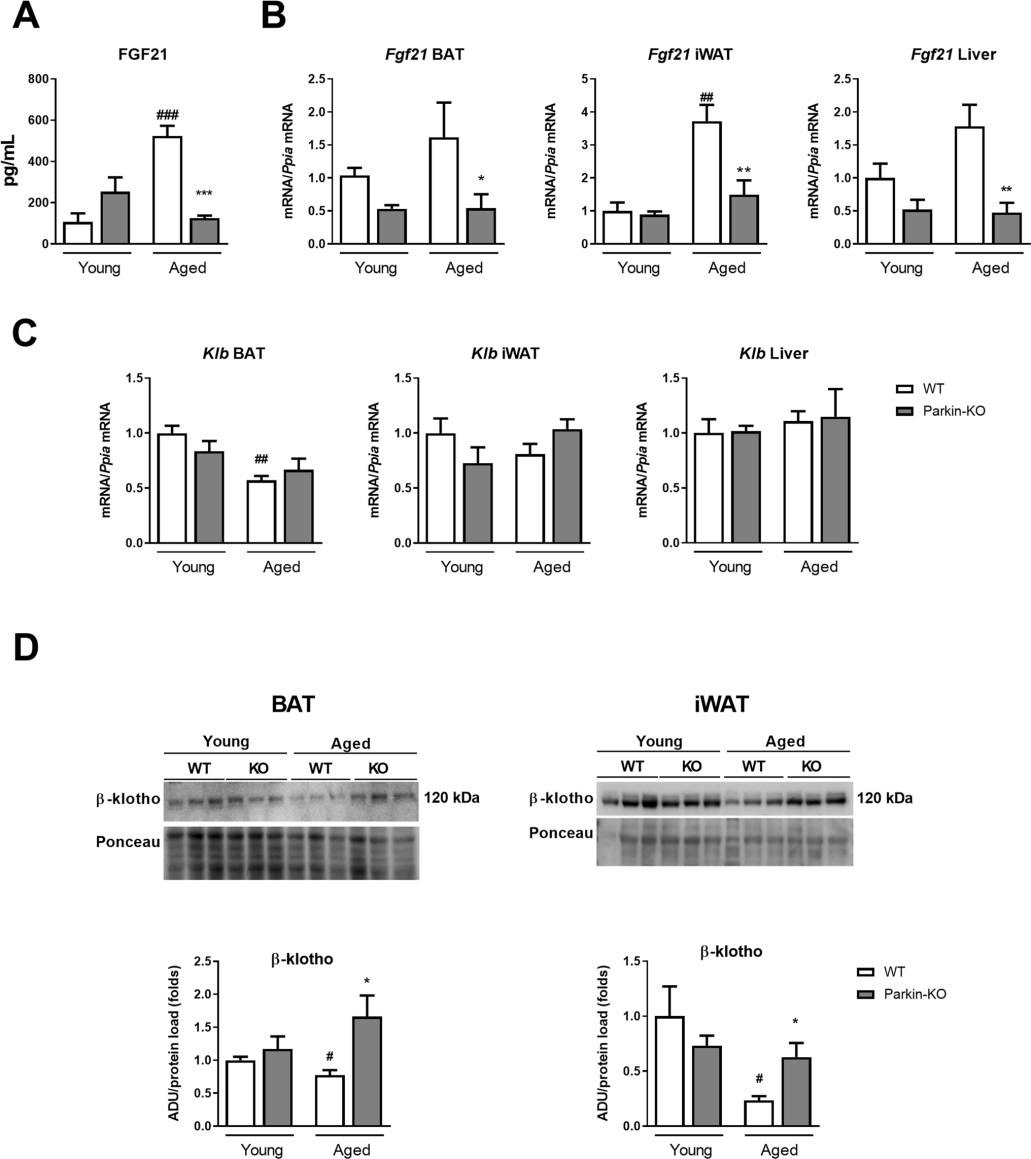
Analysis of FGF21 levels, FGF21 and β-klotho expression in white and brown adipose tissues, and liver, from young versus aged wild-type and Parkin-KO mice. (**A**) FGF21 circulating levels. (**B**) Quantification of mRNA levels of *Fgf21* in adipose tissues and liver. (**C**) Quantification of mRNA levels of *Klb* in adipose tissues and liver. (**D**) Quantification (bottom) and representative image of immunoblots (top) for β-klotho in brown and white adipose tissues from young versus aged wild-type and Parkin-KO mice. The bars represent means ± SEM. *N* = 3–8. (*p < 0.05, **p < 0.01, ***p < 0.001 when comparing wild-type versus Parkin-KO mice; #*p* < 0.05, ##*p* < 0.01, ###*p* < 0.001 when comparing young versus aged mice)

Our endocrine profiling of Parkin-KO mice revealed that the plasma levels of FGF21 were markedly induced in aged wild-type mice but not Parkin-KO mice (Fig. 3A). Consistent with this, analysis of FGF21 gene expression in iWAT revealed that there was an age-dependent induction of *Fgf21* mRNA levels in wild-type mice but not Parkin-KO mice (Fig. 3B). Similarly, levels of Fgf21 mRNA remained low in BAT from aged Parkin-KO mice. As the liver critically contributes to FGF21 systemic levels, we analyzed the hepatic expression of FGF21. We found a similar significant reduction in hepatic *Fgf21* mRNA levels in 16-month-old Parkin-KO mice relative to 16-month-old wild-type mice. Gene expression analysis of liver from 5-month-old and 16 month-old wild-type and Parkin-KO mice did not reveal significant changes due to Parkin gene invalidation for transcripts encoding components of gluconeogenesis, lipid metabolism, oxidative stress, and ER stress at any age (Supplemental Table 4).

We further analyzed the expression levels of the *Klb*, encoding the co-receptor β-klotho, which is key in providing cells with specific responsiveness to FGF21. For the *Klb* mRNA, we found that aging was associated with significant down-regulation in BAT, a non-significant trend to reduction in iWAT, and no change in liver (Fig. 3C). Parkin gene invalidation did not significantly modify the mRNA expression of *Klb* in adipose tissues from aged mice, although there was an increasing trend. However, at the protein level, β-klotho was significantly down-regulated with aging in BAT and iWAT in wild-type but not Parkin-KO mice; the β-klotho protein levels were significantly higher in aged Parkin-KO mice than compared to aged wild-type mice (Fig. 3D). The mRNA expression levels of *Fgfr1,* encoding FGF receptor-1*,* were unaltered due to the genotype or age in iWAT (Supplemental Table 2) whereas it was somewhat lower in aged BAT with no difference between Parkin-KO and wid-type mice (Supplemental Table 3).

## Discussion

In the present study, we found that Parkin invalidation prevents the increased adiposity and metabolic derangements associated with aging in mice. This is associated with maintenance of the browning of subcutaneous adipose tissue and suppression of the age-induced up-regulation of FGF21.

Our findings that Parkin invalidation has positive effects on metabolism and adiposity in aged mice are consistent with the results of previous studies in young mice challenged with thermogenic stimuli. These prior studies indicated that Parkin, as a component of the mitophagy machinery, contributes to repressing BAT function and promoting the “whitening” of beige adipose tissue [1, 20, 28, 32]. The relatively positive effects of Parkin suppression on aging may appear surprising at first glance, given that the preservation of active mitophagy is important for healthy aging [2]. Our data did not suggest that loss of Parkin impaired mitochondrial function in adipose tissues; this could reflect the action of allostatic processes, which often occur in loss-of-function models in which the targeting of a single component of a biological process (here, mitophagy) elicits intense compensatory processes that overcome any potential derangement due to the invalidation of a single actor. During the preparation of this article, it was reported [24] that Parkin gene invalidation at adipose tissues also leads to prevention of age-associated adiposity, which is consistent with our current findings. On the other hand, Parkin suppression in aged mice resulted in a reduction in food intake, which could indirectly favor a healthier metabolic status and play a role in the preservation of WAT browning. Notably, we also found that Parkin invalidation in aged mice affected preferentially the browning of WAT rather than BAT activity. There are multiple previous reports indicating a higher responsiveness of WAT browning rather than BAT thermogenic activity in experimental models challenging adaptive thermogenesis [26]. This has been attributed to a particular relevance of the regulation of WAT browning in modulating thermogenic adaptation to some metabolic conditions [26, 35] and, perhaps, to the existence of distinct non-UCP1-mediated thermogenic processes in WAT [4].

In addition to global beneficial effects on adipose and insulin resistance, the most marked effect found for Parkin invalidation across aging was suppression of the age-related induction of FGF21, which was noted at the gene expression level in adipose tissues and liver, as well as for systemic FGF21 levels. The age-related increase in FGF21 levels has been noted in humans [15] and rodent models [12], but its biological significance is not fully understood. Given that FGF21 has been proposed as an anti-aging factor [33] and is known to have beneficial effects on metabolism and adiposity [13], its age-related increase might be viewed as a mechanism that attempts to minimize aging-associated derangements. However, the results from studies performed in populations of elderly individuals indicate that there is an inverse association between FGF21 levels and longevity [30]. On the other hand, the age-associated increase in FGF21 levels is reminiscent of that occurring in obesity, suggesting that aging may be associated with a progressive loss of FGF21 responsiveness in tissues, giving rise to a reactive FGF21 up-regulation in a so-called “FGF21 resistance” scenario [10]. This possibility would be consistent with the observation of age-related down-regulation of β-klotho, the key factor for FGF21 responsiveness in adipose tissues [25], suggesting a scenario wherein sensitivity to FGF21 decreases with age in mice. However, the concept of FGF21-resistance is controversial [22] and recent data do not support the idea that an FGF21-resistant state is acquired with age, at least in humans [33]. In any case, the prevention of age-related increases in FGF21 should be viewed as a beneficial metabolic effect of Parkin invalidation. Further research is needed to elucidate the mechanisms, whether direct or indirect, by which the lack of Parkin leads to the down-regulation of FGF21. Considering that mitochondrial oxidative stress and/or endoplasmic reticulum (ER) stress are known to induce FGF21 synthesis in adipose tissues [3, 18], the FGF21 down-regulation observed in Parkin-KO mice is consistent with a lack of mitochondrial dysfunction in these mice, as shown by unaltered expression of marker genes for mitochondrial oxidative stress ROS and ER stress in Parkin-KO mice at any age.

Our data indicate that Parkin invalidation prevents aging-associated β-klotho down-regulation and FGF21 up-regulation. The mechanisms by which lack of Parkin prevents the aging-associated repression of β-klotho (and, accordingly, may preserve FGF21 sensitivity) deserve further research. Pro-inflammatory signals are major repressors of β-klotho expression in adipose tissues [9], and our current data indicate that pro-inflammatory cytokines are down-regulated in WAT from aged Parkin-KO mice; this, therefore, may underlie the observed maintenance of β-klotho levels. Moreover, it cannot be ruled out that preserved levels of β-klotho could maintain the sensitivity to FGF21 and constitute a primary event elicited by Parkin invalidation that reduces age-associated FGF21 secretion. Considering the important role of FGF21 in the browning of subcutaneous WAT, the maintenance of some extent of browning in Parkin-KO mice may also be secondary to maintenance of FGF21 signaling. In any case, the mechanisms by which lack of Parkin prevents the aging-associated repression of β-klotho (and, accordingly, may preserve FGF21 sensitivity) deserve further research. Our findings on FGF21 alterations in aging-associated adipose thermogenic decline do not rule out the potential role of other regulatory factors of adipose browning. The number of potential effectors of adaptive WAT browning is growingly extensive, among them thyroid hormones, bile acids and several peptidic endocrine actors [21–23], so further research will be needed to identify their potential role in the response to Parkin invalidation. Moreover, the use of a whole-body Parkin KO model in our study and the signs of altered eating behavior also suggest that centrally-mediated mechanisms may contribute to the observed alterations in adipose plasticity in aged mice. Parkin is widely expressed in brain, including hypothalamus [14] and some reports point to a relevant role of Parkin in the mitophagy processes in hypothalamic regions [34], key to the control of energy balance and thermogenic adipose tissue plasticity [5]. Further research will be needed to explore this possibility and their interplay with the FGF21 system.

## Conclusions

Collectively, the present data highlight the beneficial metabolic effects that, along with WAT browning, occur with aging in Parkin-KO mice. The effects of Parkin on the FGF21 system may be relevant to the systemic beneficial effects that Parkin suppression can have on aging.

### Supplementary Information

Below is the link to the electronic supplementary material.

Supplementary file1 (DOCX 21.8 KB)

Supplementary figure 1. Low resolution image (PNG 2.10 mb)

High resolution image (TIF 8.08 MB)

## Data Availability

The raw data supporting the conclusions of this article will be made available by the authors, without undue reservation.
