# Supplementary material for: Parkin depletion prevents the age-related alterations in the FGF21 system and the decline in white adipose tissue thermogenic function in mice
Source: J Physiol Biochem. 2023 Nov 2;80(1):41–51. doi: 10.1007/s13105-023-00977-x (PMC10808413; doi:10.1007/s13105-023-00977-x)
Supplement: Supplementary file 1 — Supplementary file1 (DOCX 21.8 KB) [file 13105_2023_977_MOESM1_ESM.docx]

**Supplementary Table 1:** TaqMan assays in qRT-PCR analysis of RNA transcript levels.

**TaqMan probes**

| **Primer name** | **Gene symbol** | **Catalogue number** | **Amplicon length (bp)** | **Location (exon boundary)** |
| --- | --- | --- | --- | --- |
| **Reference Gene** |  |  |  |  |
| Parkin | *Park2* | Mm01323528_m1 | 71 | 7-8 |
| Peptidylprolyl isomerase A | *Ppia* | Mm 02342430_gl | 148 | 4-5 |
| Uncoupling protein 1 | *Ucp1* | Mm 00494069_m1 | 95 | 1-2 |
| Iodothyronine deiodinase 2 | *Dio2* | Mm 00515664_m1 | 77 | 1-2 |
| Pparg coactivator 1 alpha | *Ppargc1a* | Mm 00447183_m1 | 104 | 5-6 |
| Cytochrome c oxidase subunit VIIa 1 | *Cox7a1* | Mm00438297_g1 | 64 | 2-3 |
| Peroxisome proliferator activated receptor alpha | *Pparg* | Mm 00440945_m1 | 105 | 6-7 |
| Leptin | *Lep* | Mm00434759_m1 | 73 | 2-3 |
| Adiponectin | *Adipoq* | Mm04933656_m1 | 77 | 2-3 |
| Tumor necrosis factor | *Tnf* | Mm00443258_m1 | 81 | 1-2 |
| Chemokine (C-C motif) ligand 2 | *Ccl2* | Mm00441242_m1 | 74 | 1-2 |
| Nitric oxide synthase 2 | *Nos2* | Mm00440502_m1 | 66 | 21-22 |
| Fibroblast growth factor 21 | *Fgf21* | Mm 00840165_g1 | 78 | 1-2 |
| Fibroblast growth factor receptor 1 | *Fgfr1* | Mm00438930_m1 | 76 | 10-11 |
| Klotho beta | *Klb* | Mm00473122_m1 | 113 | 1-2 |
| Acyl-Coenzyme A dehydrogenase, medium chain | *Acadm* | Mm01323360_g1 | 64 | 3-4 |
| Acetyl-Coenzyme A carboxylase alpha | *Acaca* | Mm01304258_m1 | 62 | 28-29 |
| Fatty acid synthase | *Fasn* | Mm00662319_m1 | 67 | 3-4 |
| Stearoyl-Coenzyme A desaturase 1 | *Scd1* | Mm00772290_m1 | 60 | 2-3 |
| Sterol regulatory element binding transcription factor 1 | *Srebf1* | Mm00550338_m1 | 62 | 1-2 |
| Mannose receptor, C type 1 | *Mrc1* | Mm01329359_m1 | 70 | 24-25 |
| Arginase | *Arg1* | Mm00475988_m1 | 65 | 1-2 |
| C-type lectin domain family 10, member A | *Clec10a* | Mm00546125_g1 | 82 | 2-3 |
| Superoxide dismutase 2 | *Sod2* | Mm01313000_m1 | 67 | 4-5 |
| Catalase | *Cat* | Mm00437992_m1 | 64 | 8-9 |
| Heat shock protein 5 | *Hspa5* | Mm00517691_m1 | 75 | 4-5 |
| Autophagy related 7 | *Atg7* | Mm00512209_m1 | 67 | 19-20 |
| Microtubule-associated protein 1 light chain 3 beta | *Map1lc3b* | Mm00782868_sH | 141 | 4-4 |
| Phosphoinositide-3-kinase, class 3 | *Pik3c3* | Mm00619489_m1 | 85 | 21-22 |
| Unc-51 like kinase 1 | *Ulk1* | Mm00437238_m1 | 92 | 5-6 |
| Solute carrier family 2 (facilitated glucose transporter), member 1 (Glut-1) | *Slc2a1* | Mm00441480_m1 | 73 | 8-9 |
| DNA-damage inducible transcript 3 (Chop-10) | *Ddit3* | Mm01135937_g1 | 92 | 2-3 |
| Phosphoenolpyruvate carboxykinase 1 | *Pck1* | Mm01247058_m1 | 61 | 8-9 |
| Glucose-6-phosphatase | *G6pc* | Mm04207416_m1 | 105 | 3-4 |
| 3-hydroxy-3-methylglutaryl-Coenzyme A synthase 2 | *Hmgcs2* | Mm00550050_m1 | 113 | 1-2 |
| cytochrome c oxidase subunit I | *Cox1* | Mm04225243_g1 | 78 | 1-1 |
| cytochrome b | *Cytb* | Mm04225271_g1 | 139 |  |
