# Supplementary figures and images for: Parkin depletion prevents the age-related alterations in the FGF21 system and the decline in white adipose tissue thermogenic function in mice
Source: J Physiol Biochem. 2023 Nov 2;80(1):41–51. doi: 10.1007/s13105-023-00977-x (PMC10808413; doi:10.1007/s13105-023-00977-x)

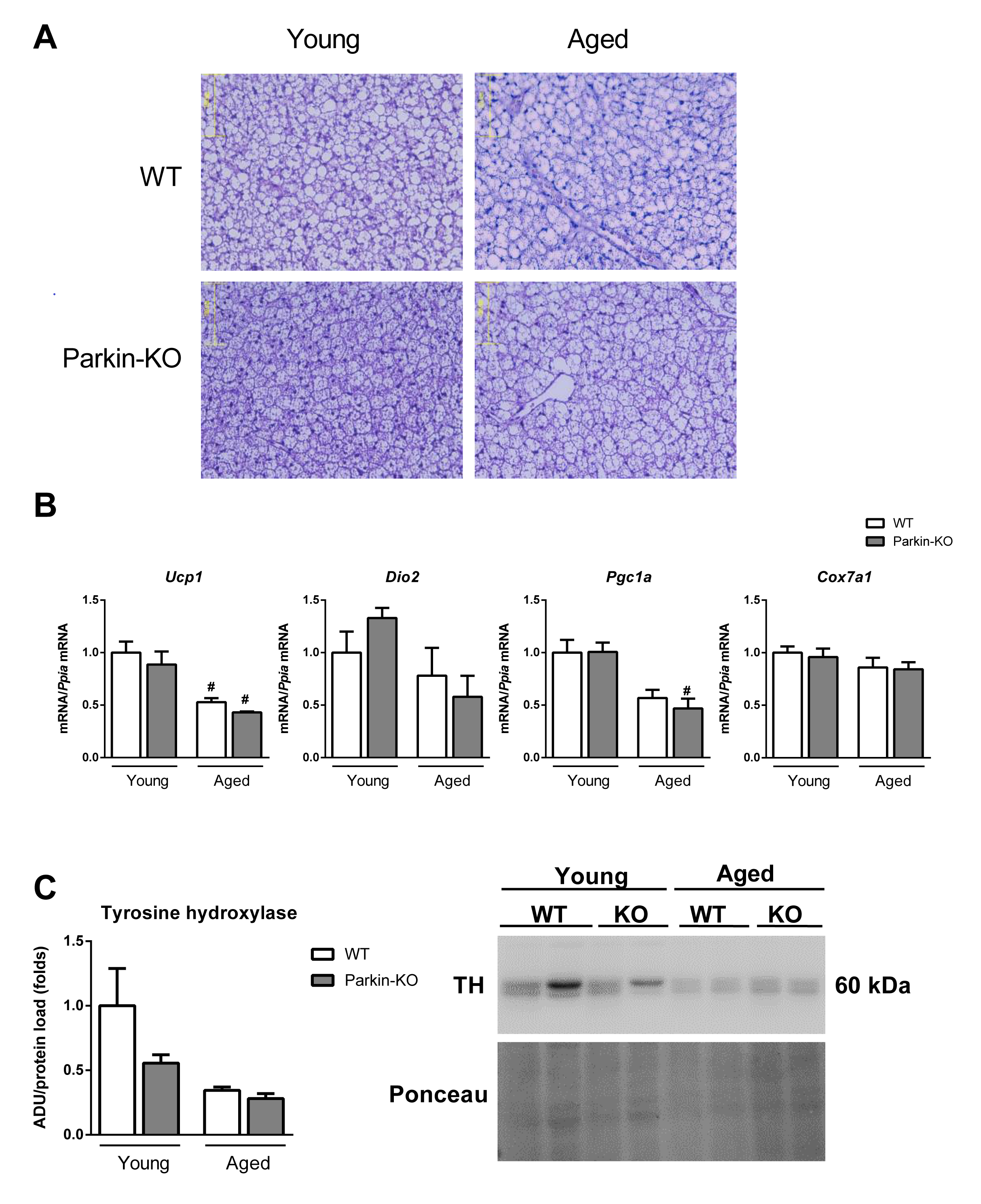

Supplement: Supplementary file 2 — Low resolution image (PNG 2.10 mb) [file 13105_2023_977_Fig3_ESM.png]

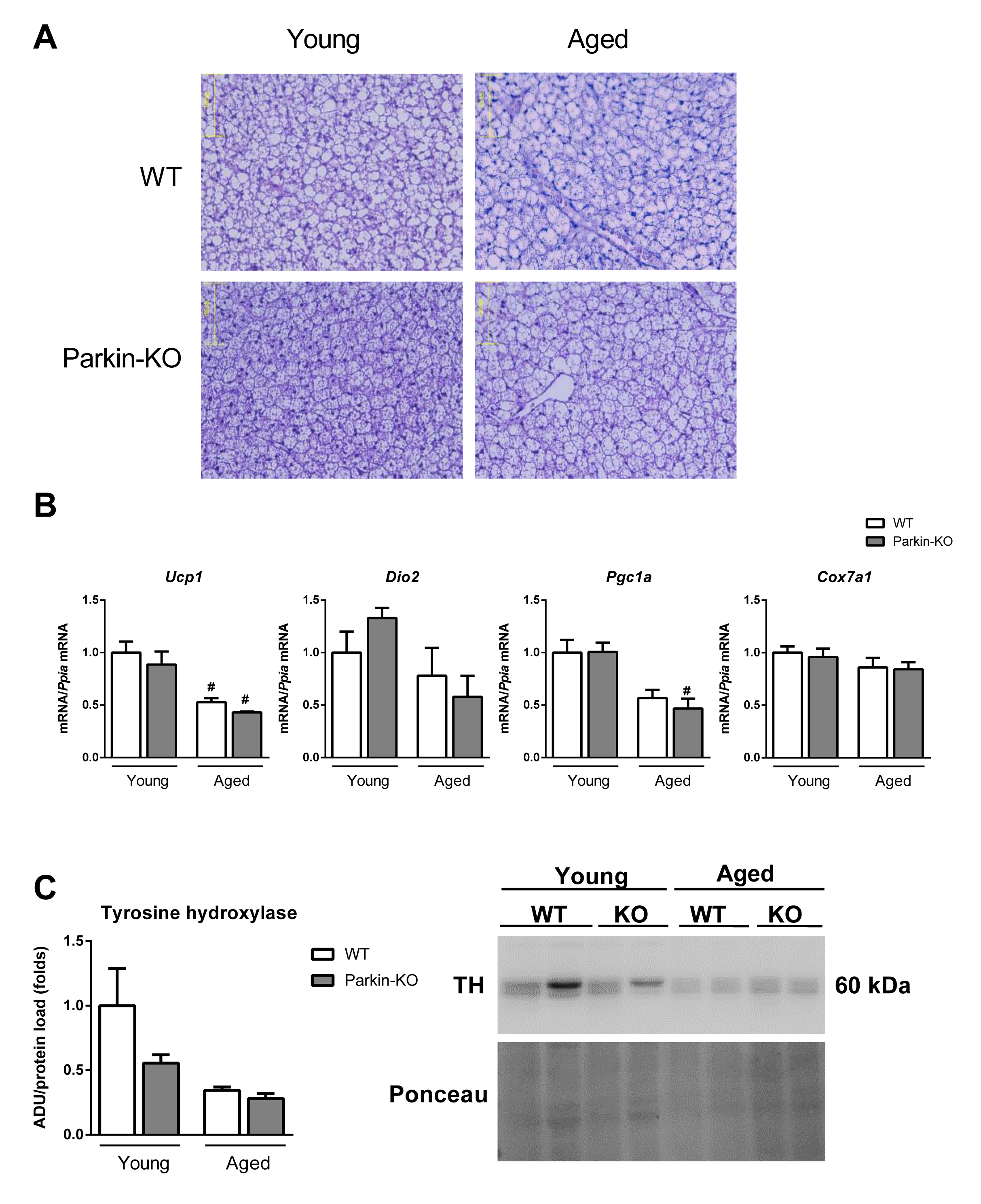

Supplement: Supplementary file 3 — High resolution image (TIF 8.08 MB) [file 13105_2023_977_MOESM2_ESM.tif]
